# High-Performing Conductive Hydrogels for Wearable Applications

**DOI:** 10.3390/gels9070549

**Published:** 2023-07-06

**Authors:** Hossein Omidian, Sumana Dey Chowdhury

**Affiliations:** Barry and Judy Silverman College of Pharmacy, Nova Southeastern University, Fort Lauderdale, FL 33328, USA; sd2236@mynsu.nova.edu

**Keywords:** conductive hydrogels, wearable sensors, performance enhancement, composite materials, multifunctionality

## Abstract

Conductive hydrogels have gained significant attention for their extensive applications in healthcare monitoring, wearable sensors, electronic devices, soft robotics, energy storage, and human–machine interfaces. To address the limitations of conductive hydrogels, researchers are focused on enhancing properties such as sensitivity, mechanical strength, electrical performance at low temperatures, stability, antibacterial properties, and conductivity. Composite materials, including nanoparticles, nanowires, polymers, and ionic liquids, are incorporated to improve the conductivity and mechanical strength. Biocompatibility and biosafety are emphasized for safe integration with biological tissues. Conductive hydrogels exhibit unique properties such as stretchability, self-healing, wet adhesion, anti-freezing, transparency, UV-shielding, and adjustable mechanical properties, making them suitable for specific applications. Researchers aim to develop multifunctional hydrogels with antibacterial characteristics, self-healing capabilities, transparency, UV-shielding, gas-sensing, and strain-sensitivity.

## 1. Introduction

Flexible conductive hydrogels have attracted significant attention due to their unique combination of mechanical flexibility, electrical conductivity, and biocompatibility, making them highly promising for a wide range of applications in healthcare monitoring, human–machine interfaces, soft robots, and wearable devices [1,2,3,4]. Understanding the structure–property correlations in these hydrogels is crucial for designing innovative products with enhanced functionality and performance in this field.

One key aspect of the structure–property correlation in flexible conductive hydrogels revolves around the molecular design and synthesis of the hydrogel matrix [1,2,3,4]. Studies emphasize the importance of selecting appropriate monomers and crosslinking agents to achieve the desired mechanical properties, flexibility, and biocompatibility. Hydrogels are typically composed of suitable monomers that undergo polymerization, with the addition of desired crosslinkers to achieve specific properties. The properties of hydrogels heavily rely on factors such as the ratio of crosslinker to monomer, the type of crosslinker used, the polymerization method employed, as well as the concentrations of initiators and monomers in the polymerization medium [5,6,7,8,9,10]. In contrast, polymer solutions with the ability to form hydrogels (known as gelators) can undergo self-assembly to form hydrogels, particularly when there is a change in temperature. These gelators can be effectively utilized in the creation of injectable hydrogel systems, which possess and offer various advantageous characteristics including thermoresponsiveness, controlled release capabilities, biocompatibility, and strong interaction with therapeutic agents [11].

The incorporation of ionic liquid segmental polyelectrolytes and conductive materials has been found to enhance the electrical conductivity of hydrogels [3,4,12]. The choice of conductive fillers also plays a crucial role in determining the electrical conductivity and sensitivity of flexible conductive hydrogels [2,13,14]. Researchers have investigated the effectiveness of various fillers such as ultralong silver nanowires, modified carbon black nanoparticles, and MXene in preserving the mechanical integrity and flexibility of the hydrogel matrix while offering high electrical conductivity [3,15]. Optimizing the filler concentration allows for achieving the desired current-carrying capacity and sensitivity of flexible electronic sensors [1].

Furthermore, the mechanical properties of flexible conductive hydrogels are essential for their applications in wearable devices and soft robotics [2,4,15]. Researchers have explored the incorporation of reinforcing agents to enhance the mechanical strength, stretchability, and stability of hydrogels [16,17,18]. Adjusting the polymer composition and tuning the freezing–thawing cycles have also proven effective in producing hydrogels with improved mechanical properties and reversible adhesiveness [19].

The interaction between the hydrogel matrix and the surrounding environment is another critical aspect of the structure–property correlation [4,20,21]. Properties such as anti-freezing, anti-drying, and moisture retention are crucial for applications in extreme environments and healthcare monitoring [17,22]. Strategies involving the incorporation of specific materials have demonstrated improved anti-freezing performance and mechanical properties [22]. Additionally, the development of hydrogel–paper patches with microfluidic channels enables real-time monitoring of electrophysiological and biochemical signals, facilitating non-invasive healthcare monitoring [23]. Environmental stability is vital for wearable sensors and electronic devices to ensure reliable performance under varying conditions [24].

Integrating responsive elements into flexible conductive hydrogels enhances their functionality in sensing and human–machine interaction applications [19,25,26]. Stimuli-responsive components have been utilized to achieve strain sensitivity, temperature response, and gesture recognition capabilities [23,25,27,28], enabling accurate detection and response to human activities, thereby advancing wearable sensors and human–computer interaction.

The double-network structure has been highlighted for its ability to enhance the mechanical properties of hydrogels. Incorporating interpenetrating polymer networks and crosslinking agents has been shown to improve stretchability, toughness, and tensile strength [29]. Moreover, achieving a combination of mechanical flexibility and high conductivity is crucial for applications in soft robotics, human–machine interaction, and artificial sensors [30].

The electrical conductivity of conductive hydrogels is vital for their integration into electronic devices and sensors. Achieving stable electrical properties and high ionic conductivity is essential [31,32]. Incorporating conductive polymers, fillers, or ion-conductive materials significantly enhances the electrical conductivity of hydrogels [31]. For example, hydrogels based on cellulose nanocrystals and carbon nanotubes show promise for strain sensors and human motion monitoring due to their self-healing properties and high conductivity [33].

Biocompatibility is a critical factor when considering the application of flexible conductive hydrogels in healthcare and wearable electronic devices. Hydrogels need to be compatible with biological systems and exhibit low cytotoxicity. Incorporating biocompatible materials enhances the biocompatibility of hydrogels [32,33].

Flexible conductive hydrogels find applications in various sensing and monitoring devices, including strain sensors, temperature sensors, and physiological signal monitoring. The properties of hydrogels significantly influence the sensitivity, linearity, response time, and durability of these sensors [30,34].

To achieve multifunctionality, conductive hydrogels often incorporate stimuli-responsive materials. For instance, thermoresponsive hydrogels can undergo reversible volume changes in response to temperature variations, enabling them to detect human motion and respond to environmental temperature changes [35].

Composite structures, such as graphene nanocomposites, have been explored to improve the mechanical and electrical properties of conductive hydrogels [31]. The addition of graphene nanosheets enhances the mechanical strength and electrical conductivity by providing a conductive network within the hydrogel matrix.

Self-healing properties have also gained significant attention in the development of flexible conductive hydrogels. These hydrogels possess the remarkable ability to autonomously repair mechanical damage, leading to a prolonged lifespan and improved durability [36].

In summary, the properties of flexible conductive hydrogels, including the mechanical properties, electrical conductivity, environmental stability, biocompatibility, and sensing capabilities, can be improved through various strategies. These advancements open up possibilities for their widespread applications in areas such as soft robotics, wearable electronics, biomedical devices, and sensor technologies. Ongoing research in this field continues to push the boundaries, unlocking new possibilities for the practical implementation of flexible conductive hydrogels.

## 2. Developments

### 2.1. Advancements in Strain-Sensitive and Conductive Hydrogels

Strain-sensitive conductive hydrogels have emerged as promising materials for the development of wearable sensors due to their unique properties, such as stretchability, flexibility, biocompatibility, and conductivity. These hydrogels enable the detection and monitoring of various human body movements, making them valuable in healthcare monitoring, biomechanics, and other applications. In this section, we will discuss several studies that highlight the advances in strain-sensitive conductive hydrogels and their applications in wearable sensors.

**Figure 1:**
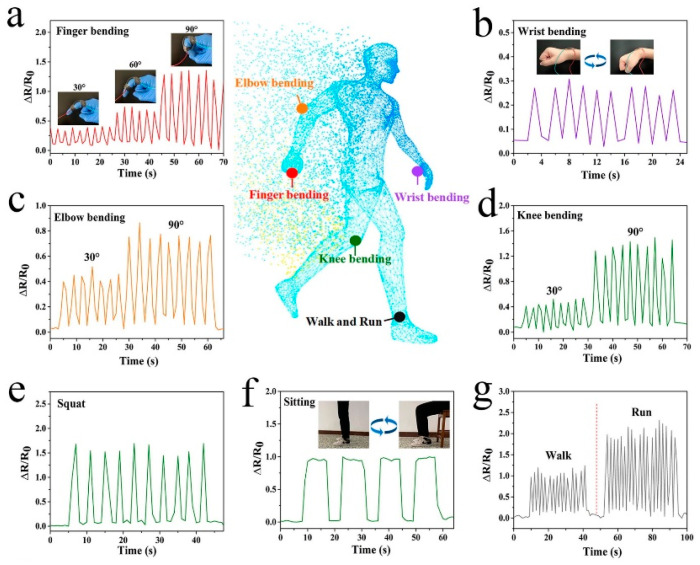
(**a**) Finger bending at different angle; (**b**) wrist bending; (**c**) elbow bending at 30° and 90°; (**d**) knee bending at 30° and 90°; (**e**) squatting; (**f**) sitting; (**g**) walking and running [1].

One study focused on the preparation of an ionic liquid segmental polyelectrolyte hydrogel, which incorporated acrylic acid (AAc), 1-vinyl-3-butylimidazolium bromide (VBIMBr), and aluminum ions (Al^3+^) [1]. The addition of ionic liquid segments improved the tensile behavior and conductivity of the hydrogel, enabling the detection of various amplitude and high-frequency limb movements. This flexible electronic sensor demonstrated stable and sensitive detection of human body movements, even in extreme environments (Figure 1).

**Figure 2:**
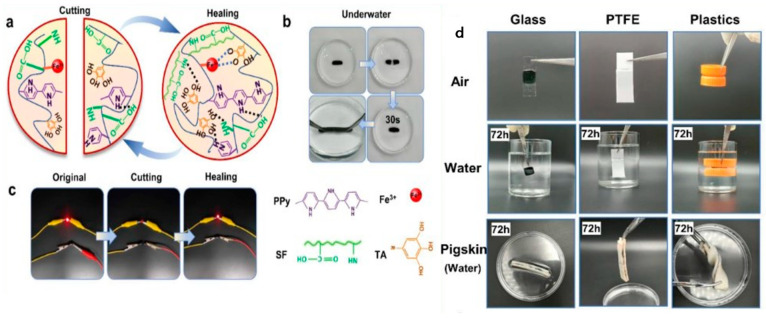
(**a**) Schematic diagram of the self-healing of SF/TA@PPy; (**b**) the underwater self-healing ability of the SF/TA@PPy hydrogel; (**c**) macroscopic image of a simple circuit composed of SF/TA@PPy-0.14M as a conductor and an LED and its electrical properties of self-healing; (**d**) adhesion of SF/TA@PPy-based conductive hydrogel to different substrates (glass, PTFE, plastic, biological tissue) in air and water [2].

Another study combined polypyrrole (PPy) with silk fibroin (SF) and tannic acid (TA) to create a conductive hydrogel for wearable strain sensors [2]. The resulting hydrogel exhibited stretchability, skin compliance, antibacterial properties, adhesiveness, and biocompatibility. It demonstrated self-healing of the mechanical, electrical, and sensing properties, making it suitable for underwater applications as well. This hydrogel adhered directly to the human body and accurately captured large- and small-strain body movements, both in air and water. Figure 2 represents the self-healing and adhesive property of the conductive hydrogel.

To further enhance the sensitivity and functionality of conductive-hydrogel-based sensors, one study developed a composite material by combining ultralong silver nanowires with modified carbon black nanoparticles [3]. This composite material was then incorporated into a poly(vinyl alcohol)/tannic acid/poly(acrylamide) (PVA/TA/PAM) hydrogel. Inspired by human neurons, the structure of the composite conductive hydrogel imitated the structure of neurons, resulting in enhanced conductivity and sensitivity. The flexible wearable sensor fabricated from this hydrogel exhibited high sensitivity, flexibility, stability, remoldability, and strain/pressure sensitivity, enabling accurate detection of various human activities.

In another study, a hybrid conductive hydrogel was developed using gamma-polyglutamic acid (PGA) and poly(3,4-ethylenedioxythiophene):poly(styrene sulfonate) (PEDOT:PSS) [4]. This hydrogel exhibited good cytocompatibility, flexibility, and conductivity. The multiple hydrogen bonding interactions endowed the hydrogel with adhesive performance, self-healing abilities, and optimal injectable properties. Skin-like sensors fabricated from this hydrogel successfully detected precise signals from human motions, enabling healthcare monitoring and understanding of biological behavior.

Electrochemical biosensors based on conductive hydrogels have also been investigated for noninvasive and continuous monitoring of various biomarkers. One study developed an electrochemical biosensor based on a PEDOT:PSS conductive hydrogel incorporated with Prussian blue nanoparticles (PBNPs) for glucose monitoring [14]. This biosensor demonstrated a low detection limit, high sensitivity, and good accuracy for glucose detection. Applied as a skin patch, it allowed for noninvasive monitoring of interstitial fluid-borne glucose, showing potential for clinically wearable noninvasive glucose monitoring in diabetics.

In the field of gel-based electronics, a structural gel composite (SGC) approach was proposed [20]. This approach involved encapsulating conductive hydrogel/MXene with a lipid gel layer (Lipogel) through in situ polymerization. The resulting SGC exhibited anti-swelling properties in aqueous environments and excellent dehydration features in open air, making it suitable for underwater mechanosensing applications. SGC-based sensors demonstrated high and stable sensitivity, enabling underwater monitoring of human motions, waterproof anti-counterfeiting applications, and tactile trajectory tracking.

Inherently antibacterial conductive hydrogels (ACGs) composed of pNIPAM and AgNWs were developed in another study [37]. These ACGs exhibited enhanced mechanical properties and excellent antibacterial activity. The ACG-based sensors demonstrated high sensitivity and captured various motion signals, enabling the development of a wearable wireless system for remote control.

Other studies focused on the development of hydrogels with adjustable mechanical properties and high sensitivity. A synergistic dual-network hydrogel and a PVA/PAA double-network hydrogel incorporated with conductive materials demonstrated outstanding conductivity and sensitivity and the ability to detect both large and delicate movements [12,19,38]. These hydrogel-based strain sensors showed potential for wearable devices and human health monitoring.

Finally, mussel-inspired hydrogels based on l-DMA-PCL were developed as potential wearable strain sensors [39]. These hydrogels exhibited reversible adhesion, tunable mechanical properties, and high strain sensitivity. They demonstrated high conductivity and sensitive responses to both large and subtle human motions, making them suitable for flexible and wearable hydrogel strain sensors in biomaterials and healthcare monitoring.

In summary, the studies presented in this section highlight the significant progress in strain-sensitive conductive hydrogels for wearable sensor applications. These hydrogels offer a range of desirable properties, including stretchability, flexibility, conductivity, biocompatibility, antibacterial activity, self-healing ability, and adhesion. They have shown promise in detecting and monitoring various human body movements, enabling advancements in healthcare monitoring, biomechanics, and other related fields.

### 2.2. Conductive Hydrogels for Wearable Sensors

In recent years, the development of conductive hydrogels for wearable sensors has gained significant attention due to their unique properties and potential applications in various fields. In this section, we will discuss several studies that highlight the advances in conductive hydrogels and their applications in wearable sensor technology.

One study focused on the preparation of an adjustable porous gelatin/polypyrrole/reduction graphene oxide (Gel/PPy/rGO) organohydrogel, which demonstrated excellent properties for wearable sensor applications [13]. This porous organohydrogel exhibited high breathability, conductivity, mechanical flexibility, anti-freezing properties, and long-term stability. The sensors fabricated from this hydrogel showed remarkable sensing sensitivity, fast response ability, and endurance. They effectively monitored a range of human activities such as finger bending, elbow bending, walking, running, and pulse beating. Additionally, the hydrogel served as flexible electrodes for accurate collection and recognition of human physiological signals, such as EMG and ECG, and as an interface between humans and machines.

Another study focused on the synthesis of an ionic conductive PVA/LNP hydrogel with excellent mechanical and functional properties [22]. By incorporating nanolignin (LNP) and aluminum chloride (AlCl_3_) into a polyvinyl alcohol (PVA) matrix, the hydrogel exhibited a rigid porous network structure, abundant ion transport channels, and high ionic conductivity. The addition of LNP improved the tensile strength, elongation at break, UV-resistance ability, and transparency of the hydrogel. This hydrogel demonstrated long-term moisturizing capability and superior anti-freezing performance, making it suitable for strain-sensing applications in detecting human motions and electrophysiological signals.

A calcium chloride/TEMPO-oxidized cellulose nanofiber-dopamine/polyacrylamide (CaCl_2_/TOCNF-DOPA/PAM) organohydrogel was fabricated with a range of desirable properties for wearable sensor applications [38]. This hydrogel exhibited high stretchability, transparency, conductivity, tissue adhesiveness, and extreme environmental tolerance. It was used as a wearable dressing for multifunctional sensors, offering potential applications in healthcare monitoring and other related fields.

In another study, a conductive PAA/PAM/MXene/TA hydrogel was developed, demonstrating good stretchability, self-healing property, and sensing performance [15]. The hydrogel exhibited good biocompatibility, making it suitable for flexible wearable sensors.

A soft stretchable conductive hydrogel composite was fabricated using alginate, carboxymethyl cellulose, polyacrylamide, and silver flakes [40]. This conductive hydrogel demonstrated phase stability, low dynamic modulus rates, high tensile strain, low resistance, and a high gauge factor. It successfully supported the operation of a light-emitting diode demonstration under mechanical deformation and measured electromyogram signals without electrical malfunction.

A multifunctional and flexible hydrogel–paper patch (HPP) was developed for simultaneous real-time monitoring of electrocardiogram (ECG) signals and biochemical signals (glucose content) in sweat during exercise [23]. Integrated with a flexible printed circuit board, the HPP served as a low-impedance ECG electrode, highly sensitive glucose sensor, and microfluidic channels for sweat collection and analysis.

A sustainable method for preparing PEDOT:PSS/PNIPAM conductive hydrogels was developed, offering potential applications in continuous monitoring of breathing patterns for sleeping patients [26]. The conductive hydrogel demonstrated resistance and transparency changes in response to temperature stimulations, providing a unique resistance–temperature relationship for wearable sensor applications.

A freezing-tolerant and robust poly(N-hydroxymethyl acrylamide)/gelatin/glycerol supramolecular conductive hydrogel with double networks was synthesized [41]. The term supramolecular refers to the structured arrangement of molecules through specific recognition patterns, without involving covalent bonds [42]. This hydrogel exhibited high strength, super extensibility, rapid self-recovery, excellent fatigue resistance, high ionic conductivity, and temperature insensitivity of the mechanical properties. It was utilized as a sensor to detect human activities, providing a promising strategy for designing stretchable conductive gels for wearable intelligent electronics.

A transparent, tough, and conductive hydrogel containing a bi-physical crosslinking network was developed using carboxymethyl chitosan (CMCS)-calcium chloride (CaCl_2_)/polyacrylamide (PAAm)/poly(N-methylol acrylamide (PNMA) [43]. This hydrogel exhibited excellent light transmittance, toughness, tensile strength, breaking strain, elastic modulus, and strain-sensing performance. It was suitable for body-surface wearable devices to monitor body joint movements and has potential applications in intelligent health monitoring systems and implantable soft electronics.

**Figure 3:**
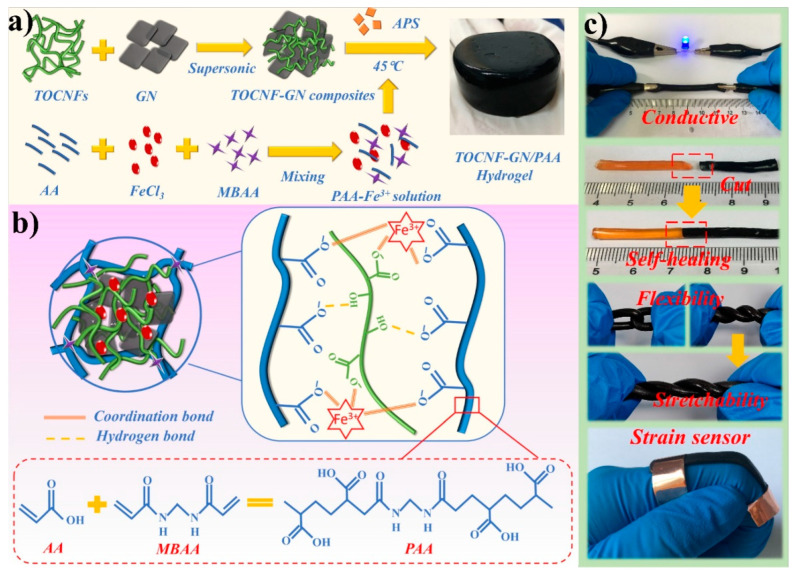
(**a**,**b**) Preparation process and formation mechanism of the TOCNF-GN/PAA composite hydrogels; (**c**) conductivity, self-healing, flexibility, and stretchability study result of composite hydrogels [18].

A composite multifunctional hydrogel for flexible sensors plays a vital role in conductive hydrogels. To compose this, TEMPO-oxidized cellulose nanofibers (TOCNFs)-graphene (tunicate cellulose nanofibrils-graphene nanosheets) nanocomposites dispersed into polyacrylic acid (PAA) hydrogel through an in situ free radical polymerization. The resulting hydrogels possess mechanical strength, electrical conductivity, self-healing capability, and adhesion to various surfaces (Figure 3).

Lastly, a multifunctional composite self-adhesive conductive hydrogel was designed, offering high stretchability, rapid self-recovery, anti-fatigue, and self-healing properties [44]. The hydrogel sensors showed a broad strain window, stretching sensitivity, and stable sensing performance for real-time monitoring of large and subtle deformations. This work provided a method to construct self-adhesive and self-healing hydrogel sensors for versatile applications in electronic skin and healthcare monitoring.

In conclusion, the aforementioned studies demonstrated significant advancements in conductive hydrogels for wearable sensor applications. These hydrogels possess various desirable properties, including high breathability, conductivity, mechanical flexibility, anti-freezing properties, long-term stability, high stretchability, transparency, and tissue adhesiveness. They have shown excellent sensing performance, enabling the monitoring of human activities, the detection of electrophysiological signals, and the collection of biochemical data. These findings pave the way for the development of innovative wearable sensors in healthcare monitoring, intelligent electronics, and other related fields.

### 2.3. Sustainable Methods for Preparing Conductive Hydrogels

Sustainable methods for preparing conductive hydrogels have emerged as a promising avenue for developing flexible and durable materials for wearable electronic devices. These materials find applications in various fields, including monitoring breathing patterns, human body motion detection, stretchable electronics, soft robots, precise body movement monitoring, healthcare monitoring, and human–machine interfaces.

One sustainable approach involved fabricating a natural-polymer-based conductive hydrogel with favorable mechanical properties, good adhesive performance, excellent fatigue resistance, and stable conductivity [21]. The hierarchical porous structure of the hydrogel, formed through the hydrogen bonding between tannic acid (TA) and chitosan (CS), enabled adjustable mechanical properties and good stretchability. The hydrogel exhibited stable conductivity, high stretching sensitivity, a rapid response time, and excellent durability, making it suitable for strain sensors to monitor human exercise behavior and physiological signals. It also provided dual-sensory performance for both temperature and strain deformation.

Another study focused on a super-stretchable, adhesive, and conductive nanocomposite hydrogel fabricated through a one-pot process involving ball milling and blending [28]. The hydrogel demonstrated super-stretchability, excellent tensile stress, fatigue resistance, and self-recovery ability due to multiple crosslinked network structures. It exhibited outstanding conductivity stability, fast response, durability, repeatability, and adhesion to various materials. The hydrogel-based strain sensors showed high sensitivity, stability, and action-recognition ability, suitable for wearable sensors in human body motion detection.

The development of a conductive hydrogel composed of silk fibroin and polypyrrole (PPy) through in situ polymerization was explored [45]. The silk-PPy hydrogel demonstrated high conductivity and responsive behavior. Flexible and wearable strain sensors based on the hydrogel showed good sensitivity, reproducibility, and stability, making them suitable for monitoring various body movements. The hybridization of biomaterials and conducting polymers allowed for multifunctionality in the conductive hydrogels, advancing wearable electronics.

Nanoparticle-enhanced polyacrylamide/hydroxypropyl guar gum/acryloyl-grafted chitosan quaternary ammonium salt/calcium ions/SiO_2_ nanoparticles (PHC/Ca^2+^/SiO_2_ NPs) conductive hydrogels were prepared [46]. The hydrogels exhibited good conductivity, toughness, stretchability, self-recovery, and fatigue resistance. They demonstrated a high maximum gauge factor, wide detectable strain range, fast response and recovery time, negligible hysteresis, and good response stability. The hydrogel was utilized to monitor human body movements such as wrist bending and pulse tracking.

In another study, a fast healable and shape memory electro-conductive hydrogel (ECH) was developed by incorporating cellulose nanocrystal-grafted phenylboronic acid (CNCs-ABA) and multiwalled carbon nanotubes (MWCNTs) into polyvinyl alcohol (PVA) [33]. The hydrogel exhibited fast healing and shape memory properties, excellent biocompatibility, remarkable mechanical properties, and enhanced conductivity. It functioned as a strain sensor for detecting human motion with superior biocompatibility and fast resistance response to applied strain, suitable for human health management.

To improve electrophysiological signal recordings on the human body surface, microneedle electrode arrays (MEAs) were fabricated [47]. The MEAs showed lower and more-stable interface impedance compared to dry electrode arrays, particularly under unstable pressures. They achieved lower noise energy, a higher signal-to-noise ratio, and higher motion-classification accuracy for electromyography (EMG) recordings. The MEA also enabled high-quality electrocardiography (ECG) recordings with accurate R-peak extraction. The microneedles penetrated the corneum without causing discomfort, providing painless signal acquisition and fixing the electrodes during body movements. This research contributes to wearable human–machine interface technology applications.

These studies collectively demonstrate the progress in developing sustainable methods for preparing conductive hydrogels, resulting in versatile materials for wearable electronic devices. By combining desirable properties such as mechanical flexibility, adhesive performance, conductivity, stretchability, and durability, these hydrogels have shown potential for various applications in healthcare monitoring, motion detection, and human–machine interfaces.

### 2.4. Conductive Hydrogels with Unique Properties

Conductive hydrogels with unique properties have garnered significant attention due to their wide-ranging implications in various fields. This section will provide an overview of studies related to conductive hydrogels with self-healing properties, antibacterial properties, and the incorporation of various additives.

Firstly, a temperature-responsive ionic conductive hydrogel with excellent stretchability, fast temperature responsiveness, and good conductivity was developed. This hydrogel holds promise for wearable strain sensors to monitor human motions and as a wearable temperature sensor to detect fever or tissue hyperthermia [48].

In another study, a polysaccharide-based dual-network hydrogel sensor with robust mechanical strength, underwater adhesion, self-healing properties, conductivity, and sensitivity was constructed. This hydrogel strain sensor sensitively monitored human motion and could detect human movement under water, making it suitable for wet and underwater environments [27].

A bilayer structure of conductive hydrogels, consisting of a spray-coated bonding interface and two different modulus hydrogel layers, was proposed in a separate study. This bilayer hydrogel exhibited outstanding stretchability, toughness, tensile strength, and elastic modulus. Additionally, a stretchable strain sensor based on this hydrogel showed good conductivity, high sensitivity, and a wide response range. The interfacial interlocking network and patch effect of the bonding interface contributed to the stable performance of the sensor. This bilayer conductive hydrogel holds promise for stretchable electronics and wearables [49].

Conductive hydrogels, which combine the properties of hydrogels and electrical conductivity, have gained attention for their potential in flexible wearable electronic devices. These hydrogels can monitor physiological and physical signals, making them a main research direction in human–computer interaction and artificial intelligence. The development and applications of conductive hydrogels in flexible wearable electronic devices are expected to continue progressing [31].

Furthermore, a tough, flexible, self-adhesive, long-term moisturizing, and antifreezing organohydrogel was prepared by incorporating gelatin, zwitterionic poly [2-(methacryloyloxy) ethyl] dimethyl-(3-sulfopropyl) (PSBMA), MXene nanosheets, and glycerol. This organohydrogel exhibited high fracture strength, stretchability, toughness, adhesion to diverse substrates, fatigue resistance, and stability under extreme temperatures. A sensor based on this organohydrogel demonstrated the ability to monitor joint motions and changes in facial expressions, highlighting its potential for wearable sensors in various environments [17].

Another study focused on an adhesive, self-healing, and antibacterial conductive hydrogel prepared using dopamine methacrylate (DMA), methacrylatoethyl trimethyl ammonium chloride (DMC), and acrylic acid (AA). This hydrogel exhibited strong adhesion to various materials, good antibacterial properties, excellent self-healing properties, ductility, biocompatibility, and conductivity. These characteristics make the hydrogel suitable for applications in electronic skin and wearable devices for monitoring physiological activities [36].

**Figure 4:**
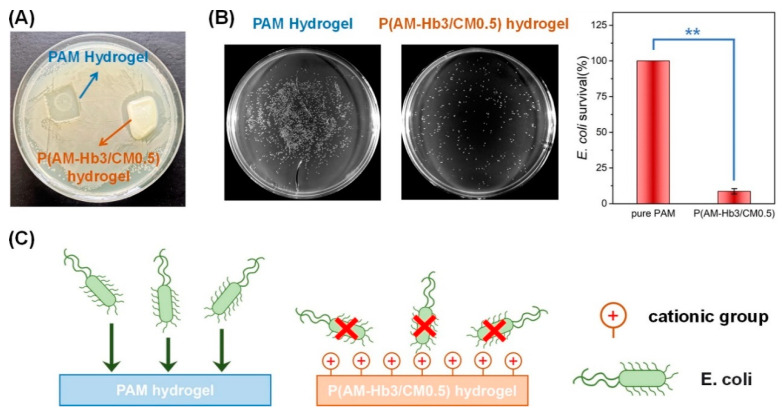
Antibacterial properties of hydrogel P(AM-Hb3/CM0.5). (**A**) Optical image showing an inhibition zone; (**B**) *E. coli* colonies and averaged survival rate after incubation with PAM or P(AM-Hb3/CM0.5) hydrogel (data reported as means ± SDs for *n* = 3 samples per group; ** represents *p* < 0.01); (**C**) mechanism of antibacterial activity [50].

Another conductive hydrogel composed of cationic micelles prepared with copolymerization of hydrophilic monomer acrylamide (AM) and hydrophobic (Hb) monomer stearyl methacrylate C18 crosslinked in the polyacrylamide (PAM) network P(AM-Hb/CM) showed antibacterial activity against *E. coli* bacteria (Figure 4) along with excellent stretchability, toughness, elasticity, and conductivity [50].

Additionally, stretchable, self-healable, and transparent gas sensors based on salt-infiltrated hydrogels were developed for high-performance NO_2_ sensing. These hydrogels demonstrated high sensitivity, a short response and recovery time, a low limit of detection, and high selectivity, stability, and conductivity. The incorporation of calcium chloride (CaCl_2_) via salt-infiltration improved the gas-sensing performance with higher sensitivity and a lower limit of detection. The salt-infiltrated hydrogel has potential for wearable electronics with gas-sensing capabilities in both anaerobic and aerobic environments [51].

Moreover, a conductive, self-healing, adhesive, and long-lasting moist MXene nanocomposite organohydrogel was prepared for wearable epidermal sensors. This hydrogel exhibited an excellent self-healing capability, self-adhesive performance, and long-lasting moisture retention. It successfully detected human motion with durable stability and could be used for wireless monitoring of human activities. This work contributes to the development of flexible, self-healing, adhesive, and long-lasting moist epidermal sensors and electronic skins for healthcare monitoring and human–machine interfaces [52].

Furthermore, a hydrophobic carbon dot nanoparticle (f-CD) mixed with polyvinyl alcohol and catechol-conjugated chitosan was used to obtain a hydrogel for pressure and vibration sensor applications. The hydrogel’s hydrophobicity influenced its mechanical performance, electronic signal acquisition, rheological reversibility, and shape recovery. Hydrogels with high hydrophobicity had a stiff structure and exhibited changes in electronic resistivity and capacitance when compressed with different forces. The hydrogel with a controlled hydrophobic–hydrophilic inner structure showed unique sensitivity and potential for applications in wearable electronic skins, healthcare monitoring, and human–computer interactions [53].

Lastly, a multifunctional hydrogel sensor was developed based on a polyvinyl alcohol substrate with poly(3,4-ethylenedioxythiophene) as the conductive filler and a glycerin/water component solvent. This hydrogel exhibited high tensile stress, large elongation, and reasonable conductivity. The glycerin/water solvent provided antifreeze and moisturizing properties to the hydrogel. This design method opens up possibilities for conductive hydrogels with antifreeze, toughness, and moisturizing properties for flexible wearable strain sensors [54].

In summary, conductive hydrogels with unique properties have immense potential in various fields such as flexible electronic devices, healthcare monitoring, biomaterials, soft robotics, gas-sensing technologies, and human–machine interfaces. These studies highlight the diverse range of properties and applications of conductive hydrogels, including self-healing capabilities, antibacterial properties, and the incorporation of various additives. Continued research and development in this field will lead to further advancements and applications of conductive hydrogels.

### 2.5. Conductive Hydrogels for Wearable Human–Machine Interfaces and Sensors

Conductive hydrogels have emerged as promising materials for wearable human–machine interfaces and sensors, offering a range of solutions to address key challenges in these fields. These hydrogels exhibit various desirable properties, including stable electrode–skin interfaces, painless signal acquisition, pressure-sensing capabilities, self-healing abilities, adhesiveness, motion detection, strain sensing, stretchability, ionic conductivity, and high sensitivity. By harnessing these properties, conductive hydrogels have the potential to advance wearable devices, human–machine interfaces, soft robotics, artificial intelligence, and sensing technologies.

To achieve mechanical resilience and conductivity, researchers have developed a double-network structure for conductive hydrogels. One such design utilized Ca^2+^ crosslinked alginate as the first dense network and ionic pair crosslinked polyzwitterion as the second loose network. This combination resulted in a hydrogel with superior mechanical properties, high stretchability, toughness, and excellent conductivity. The hydrogel also demonstrated self-healing properties, optical transparency, and adhesive characteristics, making it suitable for flexible wearable strain sensors [29].

Multifunctional conductive composite hydrogels were fabricated using poly(vinyl alcohol) (PVA), sodium alginate (SA), and tannic acid (TA). The hydrogel network was formed through borate ester bonds and hydrogen bonds. This hydrogel exhibited pH- and sugar-responsiveness, high stretchability, fast self-healing performance, and considerable conductivity. It also showed stable changes in resistance with high sensitivity, making it suitable for strain sensors. The hydrogel maintained good sensing behavior even after healing, expanding the possibilities for biocompatible polymer-based hydrogels and promoting hydrogel sensor applications [32].

A stretching/competitively coordinating/releasing (SCR) strategy was employed to prepare a self-buckled polyacrylamide/alginate hydrogel (SPAH) with highly stretchable and healable properties. The SPAH hydrogel served as an ionic conductor in a healable skin-inspired pressure sensor. It exhibited a wide pressure response range, high sensitivity to strain, a low detection limit, and excellent durability. The wearable SPAH pressure sensor successfully monitored various human motions, showcasing its potential for full-range and sophisticated motion monitoring in applications such as human–machine interfaces, soft robotics, and artificial intelligence [55].

Hydrogels with adhesiveness, toughness, self-healing, and anti-swelling properties were prepared by incorporating 2-hydroxypropyltrimethyl ammonium chloride chitosan (HACC) into the polyacrylic acid/ferric ionic (PAA/Fe^3+^) crosslinking system. These hydrogels exhibited excellent mechanical properties, self-healing efficiency, anti-swelling properties, and adhesiveness. The introduction of iron ions provided conductivity, enabling their use as flexible sensors for motion monitoring. This strategy expands the application of hydrogel-based sensors [56].

A mussel-inspired conductive hydrogel (HAC-B-PAM) was prepared using polyacrylamide (PAM), dopamine-functionalized hyaluronic acid (HAC), borax, Li^+^, and Na^+^. The hydrogel exhibited excellent stretchability, high tensile toughness, self-adhesive properties, and self-healing properties. It was utilized to fabricate a strain sensor capable of detecting human body motion. The hydrogel can be assembled into flexible wearable devices, finding applications in electronic skin and soft robotics [57].

In the realm of energy storage and wearable applications, flexible symmetric supercapacitors (FSSs) composed of solid electrolyte and electrode layers have gained attention. These devices utilize functional materials in flexible solid electrolytes and electrodes and require interface analysis and construction. Furthermore, they find applications such as high-energy-density power sources, actuators, and sensors. Rational designs of materials, device dimensions, and ion migration kinetics contribute to the multifunctionality of FSS devices, with nanomaterials and nanoscience offering opportunities for further improvement and wider adoption in wearable applications [58].

A freezing-tolerant, high-sensitivity, and durable strain and pressure sensor was constructed from a conductive chitosan-poly(acrylamide-co-acrylic acid) double-network hydrogel with dual-dynamic crosslinks. This hydrogel exhibited remarkable tensile performance, supercompressibility, elasticity, self-recovery capacity, fatigue resistance, excellent conductivity, and freezing tolerance. The sensor demonstrated cycling stability and durability in detecting pressure, various deformations, and human motions, even at low temperatures. It showcased superior sensitivity to strain and pressure compared to other organohydrogel and hydrogel sensors, providing a platform for high-sensitivity strain and pressure hydrogel sensors with wide temperature range capabilities [59].

A glycogen-based elastic, self-healable, and conductive hydrogel was developed as a strain sensor for soft electronic skin (e-skin) applications. The hydrogel exhibited self-healing efficiency, fracture stress, elongation at break, fracture toughness, and electrical conductivity. Its potential applications include wearable strain sensors, prosthetics, soft robotics, and health-monitoring systems. The utilization of natural-polymer-based hydrogels offers new possibilities for the fabrication of elastic and self-healable sensors [60].

A super-stretchable and recoverable ionic conductive hydrogel (SA-Zn) was designed as a stretchable sensor for human body motion detection. The SA-Zn hydrogel demonstrated outstanding stretchability and shape self-recovery ability. An assembled wireless wearable stretchable sensor (SA-Zn-W) highlighted excellent sensitivity, fast response, effective identification, and stable electromechanical repeatability in transforming human body motion into electrical signals. This technology provides a promising solution for remote and effective detection of human body motion [61].

An intelligent zwitterionic ionic conductive hydrogel (SAA) with a double-network structure was developed for bionic electronic skin (e-skin) applications. The SAA hydrogel exhibited high sensitivity to strain–stress and superior sensing performance in human body motion and physiological signal response. It also possessed an excellent identification ability to multiple stimuli. The SAA hydrogel holds promise for applications in sports monitoring, human–machine interfaces, and soft robotics [62].

In summary, conductive hydrogels offer a wide range of solutions for wearable human–machine interfaces and sensors. The diverse properties and functionalities of these hydrogels, such as stable electrode–skin interfaces, painless signal acquisition, pressure sensing, self-healing abilities, adhesiveness, motion detection, strain sensing, stretchability, ionic conductivity, and high sensitivity, make them suitable for various applications in wearable devices, human–machine interfaces, soft robotics, artificial intelligence, and sensing technologies. The research efforts presented in this section highlight the advancements in the design, fabrication, and application of conductive hydrogels, paving the way for future developments in this field.

### 2.6. Integration of Conductive Hydrogels in Flexible Electronics

The integration of conductive hydrogels in flexible electronics has emerged as a promising avenue for advancing various fields, including wearable technologies, artificial intelligence, healthcare monitoring, human–machine interfaces, and soft robotics. Conductive hydrogels, developed through the incorporation of nanomaterials and composite hydrogels, offer a range of desirable properties such as flexibility, high sensitivity, mechanical strength, electrical conductivity, self-healing, self-adhesiveness, and responsiveness to multiple stimuli. These advancements have paved the way for the development of innovative applications in the aforementioned areas.

One notable study focused on the synthesis of a crosslinked chitosan quaternary ammonium salt and liquid metal (CHACC-LM) composite hydrogel for smart wearable sensors [25]. This hydrogel exhibited conductivity, antibacterial properties, electrical self-healing, and strain sensitivity. It was successfully employed as a wearable flexible sensor for monitoring human activities, showing responsiveness to different stress and temperature stimuli. Moreover, the CHACC-LM hydrogel enabled gesture recognition and human–computer interaction, expanding its potential applications.

Another study explored the development of a self-healing dual-network ion conductive hydrogel by incorporating polyacrylic acid/graphene oxide-ferric cation/chitosan (PAA/GO-Fe^3+^/CS) [30]. This hydrogel demonstrated high tensile strength, conductivity, sensitivity in a wide linear range, and long-term electrical stability. It was utilized to create a stretchable strain sensor for human motion monitoring, highlighting its potential in flexible electronic device manufacturing.

In the pursuit of high deformation tolerance and excellent fatigue resistance, researchers fabricated a semi-interpenetrating ionic conductive hydrogel (SICH) through hydrogel-network-constrained polymerization [63]. The resulting SICH exhibited complex deformation tolerance, excellent fatigue resistance, and high stretchability. It served as a high deformation-tolerant ionic conductor for capacitive/resistive bimodal ionic sensors. The wearable bimodal SICH sensor demonstrated high sensitivity, linearity, a wide response range, and durability in detecting human motions.

Hydrogel-based ionic strain sensors were further explored using a hydrogen-bonded network-densification strategy, resulting in a highly stretchable and deformation-tolerant polyelectrolyte complex hydrogel (PECH) [64]. The PECH hydrogel, composed of anionic and cationic networks, exhibited large tensile strength, high stretchability, low-temperature resistance, and heat-accelerated self-healing. This densified hydrogel served as a stretchable ionic conductor for a skin-inspired ionic strain sensor, displaying high sensitivity, a fast response time, and excellent durability even at sub-zero temperatures. It demonstrated the ability to detect and distinguish complex human motions.

Another study focused on crosslinking carboxymethyl cellulose and phytic acid to achieve mechanically robust and resilient ionic conductive hydrogels with favorable ionic conductivity [65]. These hydrogels exhibited superior sensitivity, stability, and durability in sensors, with a broad strain window for detecting human activities. The approach used in this work also showed potential for large-scale, low-cost fabrication.

A silk fibroin-based conductive hydrogel was designed with stretchability and compressibility, enabling its assembly into a strain/pressure sensor with a wide sensing range [66]. This hydrogel-based sensor successfully monitored various physical signals of the human body, exhibiting biocompatibility and positive response in a triboelectric nanogenerator. With versatile applications in health and exercise monitors, soft robots, and power sources, this multifunctional material demonstrates its potential in diverse fields.

In pursuit of rapid preparation, a poly(acrylamide) @cellulose nanocrystal/tannic acid-silver nanocomposite (NC) hydrogel was synthesized using a rapid fabrication process [67]. The resulting NC hydrogels exhibited excellent stretchability, self-adhesion, strain sensitivity, and antibacterial properties. These hydrogels were utilized to assemble flexible epidermal sensors for long-term human–machine interfacial contact, demonstrating remarkable conductivity and strain sensitivity for real-time monitoring of various human motions.

The development of functional conductive hydrogels and their potential applications in wearable/implantable electronics and cell/tissue engineering have been comprehensively reviewed [59,60,61,62,66,67,68,69,70,71]. The authors emphasized the importance of formulating hydrogels with high electrical conductivity while maintaining desirable physicochemical properties. They also discussed the incorporation of additional functions such as self-healing, shape memory, and wet adhesion into conductive hydrogels for practical applications. Conductive hydrogels are seen as crucial building blocks for bioelectronic devices in personalized healthcare and bioengineering areas.

Furthermore, a study focused on the fabrication of multifunctional conductive hydrogel/thermochromic elastomer hybrid fibers for flexible wearable strain and temperature sensors [69]. By programming the extrusion of conductive hydrogel and thermochromic elastomer as core layers via dual-core coaxial wet spinning, the resulting hybrid fibers offer a core–shell segmental structure. These fibers have the potential for various applications, including human-motion monitoring, temperature detection, and color decoration. The described strategy enables mass production and can be extended to fabricate flexible wearable devices with additional components and functions.

Finally, an ultrastretchable, tough, antifreezing, and conductive cellulose hydrogel was fabricated by grafting acrylonitrile and acrylamide copolymers onto cellulose chains [70]. This hydrogel exhibits ultrastretchability, excellent tensile strength, high elasticity, good toughness, and fatigue resistance. It also demonstrates remarkable electrical conductivity and antifreezing performance, making it suitable for wearable strain sensors that exhibit high sensitivity and stability in monitoring human activities.

In summary, the integration of conductive hydrogels in flexible electronics offers immense potential for a range of applications. The studies mentioned above highlight the development and utilization of various conductive hydrogels, highlighting their unique properties and applications in wearable sensors, human–machine interfaces, healthcare monitoring, soft robotics, and more. These advancements in conductive hydrogel technology contribute to the progress of flexible electronics, opening new avenues for innovation and improved functionality in diverse fields.

### 2.7. Functional Conductive Hydrogels with Remarkable Properties

Functional conductive hydrogels have garnered significant attention due to their remarkable properties and diverse functionalities. These hydrogels exhibit high sensitivity as strain and pressure sensors, possess nature-inspired characteristics, demonstrate super-stretchability and recoverability, and enable intelligent bionic electronic skin. With applications in wearable technology, healthcare monitoring, soft robotics, artificial intelligence, and biomimetic prostheses, these hydrogels contribute to advancements in various fields.

Natural-biopolymer-based conductive hydrogels have emerged as promising materials for wearable and stretchable sensing devices. Utilizing renewable and nontoxic biopolymers such as cellulose, chitosan, silk fibroin, and gelatin, these hydrogels offer improved mechanical properties and biocompatibility [72]. Recent progress in natural-biopolymer-based conductive hydrogels has paved the way for their applications in electrical sensing devices, while also addressing the challenges and future expectations associated with these materials.

**Figure 5:**
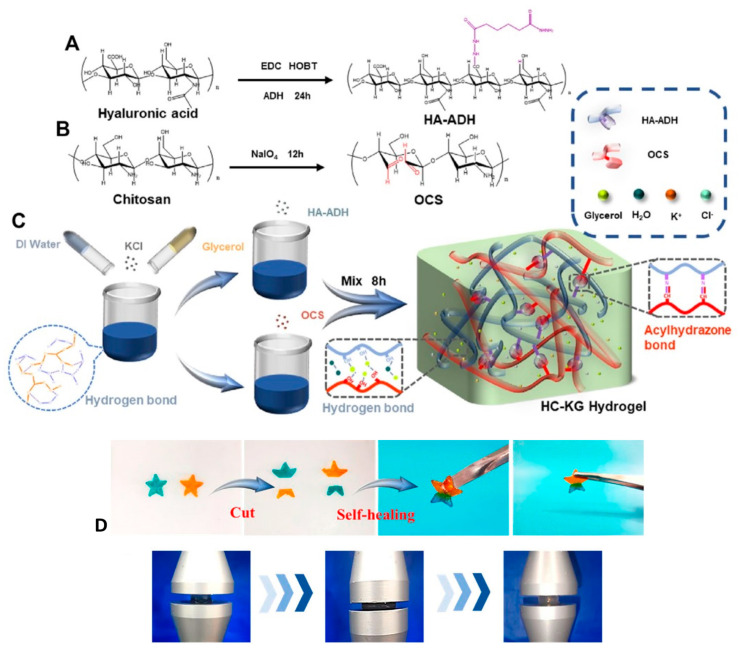
Synthesis route of (**A**) HA-ADH and (**B**) OCS (**C**) the preparation steps and the network structure of the HC-KG hydrogel; (**D**) self-healing and resilience property of the HC-KG hydrogel [73].

To enhance hydrogel functionality, including resilience, self-healing, and anti-freezing properties, natural polymers such as hydrazide-grafted hyaluronic acid (HA-ADH) and oxidized chitosan (OCS), with the presence of KCl (HC-KG hydrogel), produce a conductive hydrogel (Figure 5) [73]. This hydrogel also exhibits excellent mechanical property, as well as low water loss.

A multistimulus-responsive and multifunctional hydrogel system, composed of carboxymethyl cellulose and poly acrylic-acrylamide, demonstrates excellent elasticity, flexibility, and stable conductivity [35]. This hydrogel system exhibits remarkable capabilities in human motion detection, physiological signal response, and environmental temperature changes. By integrating a conductive hydrogel with a thermoresponsive hydrogel, a bilayer hydrogel is formed, which functions as both an actuator and a “smart” material. This hydrogel system holds promise for applications in ionic skin, smart info-windows, and soft robotics.

Another multifunctional conductive hydrogel, comprising a polyacrylamide/chitosan hybrid network, exhibits exceptional flexibility, puncture resistance, and self-healing capability [71]. With its self-adhesive behavior on various materials, this hydrogel serves as a soft human-motion sensor for real-time and accurate detection of diverse human activities. The hydrogel’s flexible, self-adhesive, self-healing, and conductive properties make it suitable for artificial intelligence, soft robots, biomimetic prostheses, and personal healthcare applications.

A hybrid hydrogel system composed of poly(N-isopropylacrylamide), poly(vinyl alcohol), sodium tetraborate decahydrate, and poly(sodium acrylate) enables the simultaneous monitoring of touch points and temperature [74]. This system demonstrates a fast response time, a temperature coefficient of resistance, and potential applications in temperature-dependent soft electronics.

Researchers have developed a highly stretchable, self-healing, and electro-conductive hydrogel with a hierarchically triple-network structure [75]. This hydrogel combines TEMPO-oxidized cellulose nanofibrils, polyacrylic acid hydrogel, and polypyrrole conductive networks, resulting in enhanced mechanical stretchability, viscoelasticity, self-healing ability, and ideal electro-conductivity. The hydrogel’s strain sensor exhibits sensitive and stable current response for monitoring human movements, making it suitable for wearable electronics.

A multifunctional conductive polymer hydrogel, incorporating multiple hydrogen-bonding 2-ureido-4[1-11]-pyrimidinone (UPy) groups as crosslinking points, demonstrates high conductivity, excellent stretchability, injectability, and rapid self-healing capability [76]. This hydrogel shows a linear response to external strain, reliable detection of human motions, and the ability to be molded into different shapes. The combination of supramolecular chemistry and conducting polymers opens up new possibilities for advanced functional materials in 3D printing, wearable devices, and flexible electronics.

To develop wearable thermoelectric-energy-harvesting fibers, researchers have produced a highly conductive p-type PEDOT:PSS fiber through a gelation process [77]. A post-treatment using organic solvents tripled the fiber’s electrical conductivity while only slightly decreasing the Seebeck coefficient, resulting in an improved thermoelectric power factor. The authors assembled a p-n-type thermoelectric device using PEDOT:PSS fibers and carbon nanotube fibers, demonstrating viable output voltage and power density for wearable energy harvesting.

A strategy for fabricating flexible, anti-fatigue, and conductive cotton fabrics involves a hierarchical coating structure comprising an l-cysteine binder, silver nanoparticles (Ag NPs), and a conductive hydrogel coating [78]. This coating structure enhances the cotton fabric’s affinity for Ag NPs, resulting in electrically conductive fabrics with high stability under deformation, suitable for smart textiles.

Lastly, a highly sensitive, low-cost, wearable pressure sensor based on conductive single-walled carbon nanotube/alginate hydrogel spheres has been developed [79]. This sensor operates on the piezoresistivity of the conductive spheres and the sphere–electrode contact, enabling successful monitoring of wrist pulse, throat muscle motion, and external pressure distribution.

In conclusion, functional conductive hydrogels with remarkable properties and functionalities have gained significant attention in recent years. These hydrogels find applications in a wide range of fields, including wearable technology, healthcare monitoring, soft robotics, artificial intelligence, and biomimetic prostheses. The studies mentioned above represent significant advancements in the development and application of functional conductive hydrogels, contributing to the progress of these fields and opening new avenues for innovation and improved functionality. Table 1 outlines the diverse strategies investigated for developing flexible conductive hydrogels suitable for a variety of applications.

## 3. Perspective

The field of strain-sensitive and conductive hydrogels has experienced significant advancements with the development of novel materials and approaches [1,2,3,4,13,14,16,20,22,37]. These hydrogels offer unique properties and functionalities suitable for a range of applications in healthcare monitoring, wearable electronics, and biomedical fields.

Conductive hydrogels for wearable sensors have made notable progress in the development of various materials and approaches [12,15,19,21,23,25,27,38,40,48]. These advancements have the potential to revolutionize the field by providing practical and reliable solutions for wearable electronics, enhancing sensor performance and reliability, and advancing healthcare and fitness management.

Sustainable methods for preparing conductive hydrogels have been developed, contributing to the production of flexible and durable materials for wearable electronic devices [26,28,31,45,49,80]. These materials find applications in monitoring breathing patterns, human body motion detection, stretchable electronics, soft robots, precise body movement monitoring, healthcare monitoring, and human–machine interfaces.

The development of conductive hydrogels with self-healing properties, antibacterial properties, and incorporating various additives has significant implications for flexible electronic devices, healthcare monitoring, biomaterials, soft robotics, gas-sensing technologies, and human–machine interfaces [24,30,33,34,36,39,41,43,46,51].

Conductive hydrogels for wearable human–machine interfaces and sensors offer solutions for stable electrode–skin interfaces, painless signal acquisition, pressure sensing, self-healing, adhesiveness, motion detection, strain sensing, stretchability, ionic conductivity, and high sensitivity [47,55,56,63,64,65,72,81,82,83]. These hydrogels contribute to advancements in wearable devices, human–machine interfaces, soft robotics, artificial intelligence, and sensing technologies.

The integration of conductive hydrogels with thermoresponsive hydrogels, the development of composite hydrogels, and the utilization of nanomaterials in flexible electronics offer flexibility, high sensitivity, mechanical strength, electrical conductivity, self-healing, self-adhesiveness, and responsiveness to multiple stimuli [18,35,44,52,53,54,57,58,84,85]. These advancements drive the progress of wearable technologies, artificial intelligence, healthcare monitoring, human–machine interfaces, and soft robotics.

High-sensitivity strain and pressure hydrogel sensors, nature-inspired glycogen-based hydrogels, super-stretchable and recoverable hydrogel-based sensors, intelligent zwitterionic hydrogel-based bionic e-skin, and other functional conductive hydrogels offer remarkable properties and functionalities [59,60,61,62,66,67,68,69,70,71]. These hydrogels find applications in wearable technology, healthcare monitoring, soft robotics, artificial intelligence, and biomimetic prostheses, contributing to advancements in these fields.

## 4. Conclusions

In conclusion, the utilization of conductive hydrogel polymers involves several strategies and considerations. The combination of conductive polymers with other materials, the enhancement of mechanical properties, the incorporation of multiple components, crosslinking and interpenetrating networks, the utilization of nanocomposites, advanced manufacturing techniques, integration with electronic components, and biomedical applications are key aspects to consider in the design and development of conductive hydrogels. These strategies aim to improve conductivity, mechanical strength, flexibility, self-healing ability, and other desirable properties required for specific applications.

However, there are also challenges that need to be addressed. One of the challenges is achieving a balance between conductivity and mechanical properties, as increasing conductivity may compromise the mechanical integrity of the hydrogel. Additionally, the stability of the conductive polymers and their interaction with other components need to be carefully evaluated to ensure long-term performance. Furthermore, the scalability and cost-effectiveness of the fabrication processes are important considerations for large-scale production and commercialization of conductive hydrogels.

Despite these challenges, conductive hydrogels hold great potential for a wide range of applications. They offer advantages such as biocompatibility, antibacterial activity, transparency, and biodegradability, making them suitable for wearable sensors, biomedical devices, antibacterial materials, and tissue adhesives. Conductive hydrogels also demonstrate high sensitivity, stability, and low detection limits, enabling their use in sensing and biosensing applications.

## Figures and Tables

**Table 1:** Materials, methods, applications, and advancements in flexible conductive hydrogels.

Materials	Methods	Advantages	Applications and Advancements	Ref.
Acrylic acid (AAc), 1-vinyl-3-butylimidazolium bromide (VBIMBr), and aluminum ion (Al(3+)).	The ionic liquid segmental polyelectrolyte hydrogel is prepared through molecular design and polymer synthesis	Enhanced tensile behavior, conductivity, and flexibility	The hydrogel enables accurate and sensitive detection of human body movements, advancing healthcare and robotics.	[1]
Conductive polymers polypyrrole (PPy), tannic acid (TA) and silk fibroin	The SF/TA@PPy conductive hydrogel is constructed by introducing into the gel network by in situ polymerization	Enhanced stretchability, antibacterial properties, self-healing capabilities, and wet adhesion to various materials.	It has applications in wearable strain sensors and underwater communication systems, advancing human motion monitoring.	[2]
Ultralong silver nanowires, modified carbon black nanoparticles, poly(vinyl alcohol)(PVA), tannic acid (TA), poly(acrylamide) (PAM)	Ultralong silver nanowires and modified carbon black nanoparticles are combined with PVA/TA/PAM to create a composite conductive hydrogel.	The composite conductive hydrogel-based flexible wearable sensor possessed high sensitivity, flexibility, stability, remoldability, and strain pressure sensitivity.	The resulting flexible wearable sensor offers high sensitivity and stability, advancing healthcare and wearable technology.	[3]
A hybrid conductive hydrogel is prepared by combining gamma-polyglutamic acid (PGA) and poly (3,4-ethylenedioxythiophene): poly (styrene sulfonate) PEDOT:PSS.		Exhibiting cytocompatibility, flexibility, high conductivity, adhesive properties, self-healing abilities, and injectability,	Its properties make it suitable for skin-like sensors and wearable healthcare devices, advancing bioelectronic applications.	[4]
Gelatin/polypyrrole/reduction graphene oxide (Gel/PPy/rGO) organohydrogel	The adjustable porous is prepared through biological fermentation and salt-out crosslinking.	Exhibit high breathability, conductivity, mechanical flexibility, anti-freezing properties, and long-term stability.	It holds potential for wearable sensors and flexible electronics, advancing signal stability and sensing sensitivity.	[13]
Prussian blue nanoparticles are incorporated into a PEDOT:PSS conductive hydrogel	Fabricated by drop-coating and embedding	High sensitivity.	The biosensor enables noninvasive glucose monitoring in diabetics, advancing glucose monitoring technology.	[14]
Conductive hydrogel/MXene is encapsulated with a lipid gel layer	Synthesized by in situ polymerization.	Providing anti-swelling properties in aqueous environments and excellent dehydration features in open-air environments.	The gel-based system has applications in underwater monitoring, anti-counterfeiting, and trajectory tracking.	[20]
Aluminum chloride (AlCl_3_) into polyvinyl alcohol (PVA) matrix and lignin nanoparticle (PVA/LNP) hydrogel	Synthesized with abundant ion transport channels	Rigid porous network structure with abundant ion transport channels, improved mechanical strength, and flexibility.	The hydrogel has applications as a strain sensor and for electrophysiological signal detection, advancing functional hydrogels.	[22]
Poly(N-isopropylacrylamide) and silver nanowires	Antibacterial conductive hydrogels (ACGs) are synthesized via two-step polymerization technique	Improved mechanical properties and antibacterial activity.	ACG-based sensors have applications in bioelectronics, health monitoring, and motion detection, advancing wearable devices	[37]
Cellulose nanocrystals (CNCs) are incorporated into a hydrogel matrix for mechanically tough and ion-conductive hydrogels.	CNCs prepared using high-pressure homogenization and pretreated with a deep eutectic solvent (DES), then incorporated them into a hydrogel matrix	Excellent mechanical properties, transparency, and freezing resistance.	The hydrogels have applications in flexible electronic devices and sensors, advancing wearable and flexible electronics.	[16]
The CHACC-LM composite hydrogel	Synthesized by crosslinking chitosan quaternary ammonium salt with liquid metal	Enhanced extensibility, antibacterial properties, electrical self-healing, and strain sensitivity.	The hydrogel has applications in smart wearable sensors, monitoring human activities, and responding to stimuli.	[25]
calcium chloride, TEMPO-oxidized cellulose nanofibers, dopamine, and polyacrylamide.	Organo-hydrogel is fabricated by combining CaCl_2_/TOCNF-DOPA/PAM.	Excellent mechanical properties and tissue adhesiveness.	In harsh environments, organohydrogel can serve as a wearable dressing, offering multifunctional sensor capabilities and advancing the field of wearable sensor technology. Its primary function is to protect the skin from frostbite or burns.	[38]
PANI-P(AAm-co-AA)@Fe(3+) hydrogel	The hydrogel synthesized by combining an iron-coordinated poly(acrylamide-co-acrylic acid) network and a conductive polyaniline network. Beta-cyclodextrin also incorporated.	Produce homogeneous interpenetrating networks with regulated crosslinking density and mechanical properties.	The hydrogel has potential applications in wearable devices, health monitoring, electronic skin, and human–machine interactions.	[12]
PAA/PAM/MXene/TA	A conductive hydrogel is prepared using hydrogen bonds to introduce MXene and TA.	Exhibit good restorability and self-healing property	The hydrogel shows promise in the field of flexible wearable sensors, offering improved stability, stretchability, sensing capabilities, and biocompatibility.	[15]
PEDOT:PSS, poly (vinyl alcohol)/poly (acrylic acid) (PVA/PAA)	A conductive hydrogel strain sensor is prepared by incorporating PEDOT:PSS into a (PVA/PAA) double network hydrogel.	Tuning mechanical properties and functionalities	The strain sensor has potential applications in wearable soft electronics, with improved mechanical properties, self-healing, and high sensitivity for detecting human motions.	[19]
Conductive hydrogel composite using alginate, carboxymethyl cellulose, polyacrylamide, and silver flakes.	The conductive hydrogel composite fabricated via sol–gel transition.	Exhibit maximal tensile strain, low deformations of cyclic loading, low resistance	The composite finds applications in wearable electronics, enabling stable signal transmission and measurement during various motions.	[40]
The conductive hydrogel composed of polyvinylpyrrolidone/tannic acid/Fe(^3+^) (PVP/TA/Fe(^3+^)), N,N-methylene diacrylamide and poly(N-isopropylacrylamide-co-acrylamide) P(NIPAAm-co-AM)	The conductive hydrogel is prepared by introducing a (PVP/TA/Fe(^3+^)) crosslinked network into a N,N-methylene diacrylamide and P(NIPAAm-co-AM) network.	Enhanced stretchability and conductivity	The temperature-responsive hydrogel is suitable for flexible electronic sensors, monitoring human health, detecting motions, and measuring environmental temperature.	[48]
Dialdehyde carboxymethyl cellulose (DCMC), chitosan (CS), poly(acrylic acid) (PAA), and aluminum ions (Al(^3+^)). (DCP hydrogel)	Hydrogel sensor fabricated by forming reversible dynamic chemical bonds and physical interactions between DCMC, CS, PAA, and (Al(^3+^)).	Exhibit robust mechanical strength and good adhesive and self-healing properties.	The DCP hydrogel strain sensor can sensitively monitor human motion and show steady detection of movement underwater, opening possibilities for intelligent sensors.	[27]
Poly (3,4-ethylenedioxythiophene): poly (styrene sulfonate) (PEDOT:PSS)	The highly porous PEDOT:PSS hydrogel is self-assembled on paper fiber.	Good conductivity, hydrophilic wettability, low-impedance ECG electrode properties, and microfluidic channels for sweat collection and analysis	The HPP serves as a wearable device for real-time monitoring of ECG and biochemical signals during exercise, with applications in healthcare and fitness management.	[23]
Tannic acid (TA) into a chitosan (CS)	The conductive hydrogel is fabricated by interpenetrating tannic acid into a chitosan crosslinked network in an acidic aqueous solution.	Hierarchical porous structure	The developed hydrogel enables the development of versatile wearable sensors and soft actuators, monitoring human exercise, physiological signals, and temperature.	[21]
PEDOT:PSS/PNIPAM	The conductive hydrogels synthesized by ultrasonication-initiated NIPAM polymerization in ice bath.	It has uniform texture and good flexibility for rapid resistance	The thermosensitive conductive hydrogel can be used as a wearable sensor to monitor breathing patterns in sleeping patients, with potential for various sensing applications.	[26]
Powdered cellulose, acrylamide, potassium persulfate, N,N′-methylenebisacrylamide, N,N,N′,N′-tetramethylethylenediamine, iron chloride hexahydrate, and Potassium Chloride	Nanocomposite hydrogel is prepared through one-pot process in conjunction ball milling and blending, forming multiple crosslinked network structures.	Conductivity stability, fast response, durability, repeatability and excellent adhesion.	The nanocomposite hydrogel has potential applications in wearable sensors for human body motion detection, offering high stretchability and stability for advanced wearable sensor technologies.	[28]
Hyaluronic acid (HA)and oxidized chitosan (CS), with KCl	The conductive hydrogel was prepared through a Schiff base reaction between hydrazide-grafted HA and CS, with KCl as conductive filler	Excellent anti-freezing and anti-drying property.	The conductive hydrogel finds applications in human motion monitoring, artificial skin, brain-computer interfaces, and wearable electric sensors, with enhanced mechanical properties and practical use.	[73]
Bilayer conductive hydrogel structure with spray coated PEDOT:PSS bonding interface	Interlocking interface achieved through spray coated PEDOT:PSS. low modulus hydrogel on top, and high modulus hydrogel on bottom	Exhibits good conductivity, high sensitivity, and wide response range	The bilayer conductive hydrogel has applications in stretchable electronics, soft robots, and next-generation wearables, offering improved mechanical and electrical properties for various stretchable applications.	[49]
A conductive hybrid hydrogel using pyrrole and silk fibroin	Synthesized by in situ polymerization	It shows high conductivity, high sensitivity and fast responses to corresponding conformation changes.	The integration of biomaterials and conducting polymers enables multifunctionality, making the conductive hydrogel suitable for wearable electronics and strain sensors in monitoring human motions.	[45]
Polyacrylamide (PAM),sodium carboxymethyl cellulose (CMCNa) in dimethyl sulfoxide-water binary solvent system, Zn^2+^	The double network organohydrogel is prepared through photoinitiation polymerization and complexation of transition metal ions	Produce synergistic effect on mechanical properties and conductivity	The conductive organohydrogel finds applications in soft robots, artificial sensors, energy storage devices, and more, driving advancements in various fields.	[80]
Sodium p-styrene sulfonate, acryloxyethyl trimethyl ammonium chloride, 2-azobis (2-methyl-propionamidine) dihydrochloride, D-(+)-gluconic acid δ-lactone, ethylenediaminetetraacetic acid calcium disodium salt hydrate and sodium alginate	Fabricated by one pot/two-step method. The hydrogel consists of a double network structure consisting of Ca^2+^ crosslinked alginate and ionic pair crosslinked polyzwitterion	Exhibits excellent mechanical properties, conductivity and good self-healing performance	The hydrogel serves as a flexible strain sensor, accurately detecting human motions and facilitating diverse applications in healthcare and human–machine interfaces.	[29]
The organohydrogel incorporates gelatin, zwitterionic poly [2-(methacryloyloxy) ethyl] dimethyl-(3-sulfopropyl) (PSBMA), MXene nanosheets, and glycerol		It shows good stability under −40 degrees C along with long-term moisturizing properties and antifreeze tolerance	The organohydrogel enables reliable performance in monitoring joint movements and emotional expressions, contributing to advancements in healthcare and human–machine interfaces.	[17]
Composite hydrogels using poly(vinyl alcohol) (PVA), sodium alginate (SA), and tannic acid (TA) with borax as a crosslinker	Prepared through one-pot method	This hydrogel exhibited pH- and sugar-responsiveness, high stretchability, fast self-healing performance without any external stimulus.	The hydrogel’s multifunctional properties and high sensitivity make it suitable for advancements in hydrogel-based sensors and monitoring human motions.	[32]
Polyacrylic acid/graphene oxide-ferric cation/chitosan (PAA/GO-Fe^3+^/CS)	The self-healing dual network ion conductive hydrogel is fabricated through a simple soaking strategy	High sensitivity in a wide linear range and long-term electrical stability.	The PAA/GO-Fe^3+^/CS hydrogel is suitable for flexible electronic devices, wearable sensors, and more, offering high sensitivity, stability, and broad application prospects.	[30]
Dopamine methacrylate (DMA), methacrylatoethyl trimethyl ammonium chloride (DMC), and acrylic acid (AA). PDDA hydrogel	Prepared through free-radical copolymerization of DMA, DMC, and AA.	It exhibits strong adhesion good antibacterial properties, excellent self-healing property, ductility and biocompatibility	The multifunctional PDDA hydrogel finds applications in electronic skin and wearable devices, providing advancements in monitoring physiological activities.	[36]
3,4-dihydroxyphenyl-l-alanine acrylamide-polycaprolactone (l-DMA-PCL)	The hydrogels are prepared by crosslinking l-DMA monomers using functionalized PCL via UV light	Exhibited reversible adhesion to various material	The l-DMA-PCL hydrogel strain sensor holds potential in biomaterials and healthcare monitoring, representing advancements in flexible and wearable strain sensors.	[39]
Phytic acid, polyacrylamide, chitosan	Phytic acid is incorporated into polyacrylamide/chitosan hydrogels to fabricate a highly conductive hydrogel	Exhibited excellent flexibility and great conductivity at −20 degree	The highly conductive hydrogel finds applications in flexible wearable electronics, highlighting advancements in environmental stability for wearable electronics.	[24]
Poly(N-hydroxymethyl acrylamide), gelatin, and glycerol	Conductive hydrogel synthesized using one-pot method.	High freezing tolerance, rapid self-recovery, and anti-freezing resistance.	The conductive hydrogel holds promise for wearable intelligent electronics, offering stretchability, mechanical properties, and integrated high performance.	[41]
Carboxymethyl chitosan, calcium chloride, polyacrylamide, and poly(N-methylol acrylamide)	Synthesize through in situ free radical polymerization.	Transparent and tough	The hydrogel finds applications in body-surface wearable devices for monitoring joint movements, intelligent health monitoring systems, and implantable soft electronics.	[43]
Polyacrylamide, hydroxypropyl guar gum, acryloyl-grafted chitosan quaternary ammonium salt, calcium ions, and SiO_2_ nanoparticles (PHC/Ca^2+^/SiO_2_ NPs)	Fabricated by mixing all the ingredients at different order and different temperature with stable chemical and physical hybrid crosslinking networks and reversible non-covalent interactions	It shows good conductivity, excellent toughness, high stretchability, self-recovery and good fatigue resistance	The conductive hydrogel can be used in monitoring body movements, soft robots, epidermal electronics, and human–machine interactions, contributing to advancements in these areas.	[46]
cellulose nanocrystals grafted phenylboronic add (CNCs-ABA) and multiwalled carbon nanotubes (MWCNTs), polyvinyl alcohol (PVA)	This double crosslinking network hydrogel fabricated by a two-step method. Firstly, a homogeneous suspension obtained by mixing PVA with CNCs-ABA dispersed MWCNTs suspension and after freeze thaw cycle microcrystallization crosslinked hydrogel obtained. Secondly, a dual crosslinked hydrogel developed by soaking the microcrystallization crosslinked hydrogel into 1.0 M NaOH.	Excellent shape recovery	The hydrogel holds potential for wearable strain sensors in human health management, addressing toxicity and long healing times associated with synthetic polymer-based Electro-conductive hydrogel (ECHs).	[33]
Acrylamide, methylene Bisacrylamide, K- carrageenan, ammonium persulfate	The polyacrylamide/carrageenan (PAAm/Carr) double network (DN) hydrogel was prepared by the one-pot polymerization method, incorporates calcium chloride via a salt-infiltration strategy.	Exhibit excellent selectivity of NO_2_ and self-healable property	The salt-infiltrated hydrogel is suitable for wearable electronics with gas sensing capabilities in both anaerobic and aerobic environments, offering stretchability and self-healing properties.	[51]
Tannic acid-coated hydroxyapatite nanowires (TA@HAP NWs), PVA chains, ethylene glycol (EG), and metal ions	The hydrogel prepared by hydrothermal reaction, and in-situ oxidation deposition.	High sensitivity within a wide strain range, high linearity, fast response and excellent cycle stability.	The conductive hydrogel can be applied in ionic skin devices, soft robots, and human–machine interfaces, offering advancements in sensing, UV-protection, moisture retention, and freezing resistance.	[34]
Epoxy resin, a curing agent, pure iron powders	The microneedle electrode array (MEA) is fabricated using a magnetization-induced self-assembly method.	Provide more stable interface impedance under unstable pressures	The MEA enables wearable human–machine interface technology by penetrating the corneum and reaching the epidermis for reliable electrode fixation on the body surface.	[47]
Polyacrylamide/alginate hydrogel (SPAH)	The self-buckled (SPAH) is prepared using the stretching/competitively-coordinating/releasing (SCR) strategy.	High stretchability and programmable wrinkled surfaces.	The SPAH hydrogel finds applications in full-range motion monitoring and human–machine interfaces, soft robotics, and artificial intelligence, owing to its stretchability and healability.	[55]
2-hydroxypropyltrimethyl ammonium chloride chitosan (HACC), polyacrylic acid/ferric ionic crosslinking system	A conductive hydrogel is prepared using non-covalent interactions	Exhibiting excellent mechanical properties, self-healing, anti-swelling, and adhesiveness.	The multifunctional conductive hydrogel is suitable for flexible sensors, offering unique properties such as adhesiveness, toughness, self-healing, anti-swelling, and conductivity.	[56]
1-butyl-3-vinylimidazole tetrafluoroborate and acrylic acid in polyethylene oxide aqueous solution	The semi-interpenetrating ionic conductive hydrogel (SICH) is fabricated through hydrogel-network constrained polymerization	Enabling stretchability and compressibility with immediate recovery	The SICH hydrogel has potential in high deformation-tolerant ionic sensors for capacitive/resistive sensing, contributing to advancements in wearable sensor technologies.	[63]
Polyacrylic acid, chitosan and Fe^3+^	Highly stretchable and deformation-tolerant PECH hydrogel (Fe/CS/PAA) prepared by combining anionic Fe^3+^-coordinated polyacrylic acid network (Fe-PAA) and cationic Fe^3+^-coordinated chitosan network (Fe-CS) through hydrogen-bonded network densification strategy activated by salt impregnation.	Exhibiting large tensile strength, stretchability, and self-healability.	The DHB-Fe/CS/PAA hydrogel serves as a stretchable ionic conductor, particularly for skin-inspired ionic strain sensors, enabling real-time detection of complex human motions.	[64]
Acrylamide (AM), ammonium persulphate(APS), N,N′-methylenebisacrylamide(MBAA), N,N,N′,N′-tetramethylethylenedia-mine(TEMED), AlCl_3_ and Li_2_SO_4_	The strain sensor is developed using polymerization and ion exchange.	High ionic conductivity, ultra- stretchability, and superior linear dependence of strain sensitivity.	The mechanically durable and super-tough strain sensor finds applications in electronic skin, wearable sensors, and other areas requiring robust and sensitive strain detection.	[81]
Acrylic acid (AA), ammonium peroxydisulfate (APS), calcium chloride (CaCl_2_). Ice structuring proteins (ISPs)	The conductive hydrogel fabricated by one-step method.	Exhibited good flexibility, recovery and conductivity at room temperature and sub-zero temperature	The hydrogel’s low-temperature adaptability enables its use as strain and temperature sensors in cold environments, opening possibilities for cold-weather wearable devices and sensing applications.	[82]
The ionic conductive hydrogel composed of carboxymethyl cellulose and phytic acid,	Prepared through a one-pot approach	Exhibiting favorable mechanical performance and high ionic conductivity.	The hydrogel’s mechanical performance and ionic conductivity make it suitable for sensing and flexible devices, offering a broad strain window and potential for large-scale production.	[65]
The zwitterionic composite hydrogel combines waterborne polyurethanes and poly(sulfobetaine zwitterion-co-acrylamide)	Fabricated by one-pot radical copolymerization	Providing good stretchability, mechanical strength, ionic conductivity, and adhesion.	The zwitterionic hydrogel finds applications in wearable devices, particularly as strain/stress sensors for detecting human body movements and voice recognition, due to its stretchability and adhesion properties.	[83]
Carboxymethyl cellulose and polyacrylic-acrylamide	The hydrogel system fabricated with bilayer structure by integrating a conductive hydrogel and a thermoresponsive PNIPAM hydrogel	Exhibited excellent human motion detection and physiological signal response along with possessed the ability to respond to environmental temperature changes.	The multistimulus-responsive hydrogel system finds applications in ionic skin, smart info-window, and soft robotics.	[35]
The TEMPO-oxidized cellulose nanofibers (TOCNFs)-graphene (GN) nanocomposites into polyacrylic acid (PAA) hydrogel	Fabricated via in-situ free radical polymerization.	The self-healing, conductive hydrogel has potential as a strain sensor in self-healing wearable electronics.	Its high sensitivity and self-healing capabilities enable applications in health monitoring and human–machine interaction.	[18]
Graphene oxide (GO), polyvinyl alcohol (PVA) and polydopamine (PDA)	The graphene-based conductive hydrogel, synthesized using dissolution of PVA, dispersion of GO, addition of dopamine with pH adjustment, vigorous stirring, and mixing with borax solution.	Exhibit self-adhesive surface electrodes to detect human electrophysiological (ECG) signals	The self-adhesive, self-healing hydrogel has applications as a wearable sensor for continuous monitoring of human motion and physiological parameters. Its flexibility and versatility enable monitoring of various movements.	[84]
Chitosan (CS), tannic acid (TA), polyacrylic acid (PAA), and ionic crosslinker Al^3+^.		Contribution of synergistic coordination bonds and hydrogen bonds to hydrogel properties.	The multifunctional hydrogel sensors find applications in electronic skin, healthcare monitoring, and medical electrodes. Their high sensitivity and wide detection range enable versatile applications in different fields.	[44]
The MXene nanocomposite organohydrogel is composed of MXene nanosheet network, dopamine grafted sodium alginate (Alg-DA), phenylboronic acid grafted sodium alginate (Alg-PBA), polyacrylamide (PAAm), and glycerol/water.	The organohydrogel is prepared by conformal coating of MXene nanosheet network with Alg-DA, Alg-PBA and (PAAm) polymer networks using a glycerol/water binary solvent.	exhibits excellent self-healing capability, superior self-adhesive performance and long-lasting moisture retention	The MXene nanocomposite organohydrogel-based epidermal sensors have applications in personalized healthcare monitoring, human–machine interfaces, and artificial intelligence.	[52]
Carbon dot nanoparticles (f-CD) with polyvinyl alcohol (PVA) and catechol-conjugated chitosan (C-chitosan).	The hydrogel with controlled hydrophobic-hydrophilic inner structure is fabricated by mixing hydrophobic (f-CD) with PVA and C-chitosan.	Exhibit stiff structure and mechanically dependent volume transition.	The hydrogel finds applications in wearable electronic skins, real-time clinical health-care monitoring, and human–computer interactions. It offers unique sensitivity for pressure and vibration sensing.	[53]
A polyvinyl alcohol substrate with poly(3,4-ethylenedioxythiophene), glycerin/water	PEDOT is formed by in situ polymerization of EDOT in PVA/water/glycerin solution The gel fabricated via one-pot method	It offers improved antifreezing, toughness, and moisturizing properties,	The multifunctional hydrogel sensor finds applications in flexible wearable strain sensing. Also, expanding the utility of conductive hydrogels in wearable devices.	[54]
Polyacrylamide (PAM), dopamine-functionalized hyaluronic acid (HAC), borax, Li^+^, and Na^+^ are utilized to prepare the mussel-inspired conductive hydrogel (HAC-B-PAM)	Fabricated via a facile approach.	Excellent stretchability, high tensile toughness, self-adhesive properties and good self-healing properties without any stimuli at room temperature.	The mussel-inspired conductive hydrogel and the hydrogel-based strain sensor find applications in electronic skin and soft robotics. They offer superior properties compared to traditional metal conductors.	[57]
Chitosan-poly(acrylamide-co-acrylic acid)	The double-network hydrogel is fabricated by soaking a composite hydrogel in FeCl_3_ solution,	Enhancing mechanical properties and conductivity.	The freezing-tolerant and high-sensitive strain and pressure sensor finds applications in flexible electronics, wearable devices, and robotics, contributing to advancements in hydrogel sensor development.	[59]
The hydrogel is composed of a physically crosslinked biopolymer-based system with intercalated iron (III) ions and poly (acrylic acid).	The fabrication process involves synthesis, crosslinking, and incorporation of iron (III) ions.	Excellent self-healing efficiency and electrical conductivity	The glycogen-based hydrogel with elastic, self-healable, and conductive properties have applications in wearable strain sensors, prosthesis, soft robotics, and health monitoring, advancing the development of e-skin sensors.	[60]
The hydrogel (SA-Zn) is composed of a double network polymer sodium alginate, poly acrylic-acrylamide, and ZnSO_4_.	Its synthesis involves crosslinking of SA, PAA and ZnSO_4_	Exhibited outstanding stretchability and excellent shape self-recovery.	The super-stretchable and recoverable hydrogel-based sensor (SA-Zn-W) effectively detects human body motion, offering potential for wearable health-care detection and human–machine interaction.	[61]
The hydrogel (SAA) is composed of a double network structure incorporating sodium alginate, poly acrylic-acrylamide, and NaCl.	Its synthesis involves crosslinking of the hydrogel components and polymerization.	Exhibits high sensitivity to strain–stress and identify the superposed signals of multiple stimuli	The SAA hydrogel-based bionic e-skin with multiple stimuli responsiveness finds applications in sports monitoring, human–machine interfaces, and soft robotics, advancing the field of intelligent e-skin for real-world applications.	[62]
Silk fibroin, polyacrylamide, graphene oxide, and poly(3,4-ethylenedioxythiophene):poly(styrenesulfonate).	The silk fibroin-based hydrogel is assembled into a strain/pressure sensor with proportional mixing.	Biocompatible with no anaphylactic reaction on human skin.	The multifunctional silk fibroin-based hydrogel finds applications in wearable electronics, including health and exercise monitors, soft robots, and power sources, contributing to advancements in the field of wearable technology.	[66]
A versatile poly(acrylamide) @cellulose nanocrystal/tannic acid-silver nanocomposite hydrogel	Synthesized through radical polymerization.	It offers stretchability, self-adhesion, strain sensitivity, antibacterial properties, and conductivity	The poly(acrylamide) @cellulose nanocrystal/tannic acid-silver hydrogel finds applications in flexible electronic skin, biomedical devices, and soft robotics, advancing long-term human–machine interfacial contact and addressing bacterial breeding concerns.	[67]
Use of reduced-graphene-oxide-doped poly(2-acrylamido-2-methyl-l-propanesulfonic acid-co-acrylamide) hydrogel and thermochromic elastomer as strain-sensitive and thermosensitive materials.	Multifunctional conductive hydrogel/thermochromic elastomer hybrid fibers are fabricated using a wet-spinning method.	They serve as flexible wearable strain and temperature sensors.	The hybrid fibers with core-shell segmental structure offer diverse functionalities and scalability. They contribute to the development of flexible wearable devices with components like transistors, sensors, displays, and batteries.	[69]
Acrylonitrile, acrylamide and zinc chloride	An ultrastretchable, tough, and conductive cellulose hydrogel is synthesized by grafting acrylonitrile and acrylamide copolymers onto cellulose chains in the presence of zinc chloride	It is suitable for wearable strain sensors, even in subzero temperatures.	The conductive cellulose hydrogel exhibits excellent antifreezing and mechanical properties, enabling reliable and sensitive monitoring of human activities in flexible electronics and wearable technology.	[70]
Polyacrylamide (PAAm)/chitosan (CS), carboxyl-functionalized multi-walled carbon nanotubes (c-MWCNTs).	A multifunctional conductive hydrogel is fabricated by hybrid network crosslinked with hydrophobic associations.	It offers flexibility, self-healing, and conductivity.	The conductive hydrogel-based strain sensor finds applications in artificial intelligence, soft robots, biomimetic prostheses, and personal healthcare, contributing to wearable technology and accurate detection of human motions in health monitoring systems.	[71]
Poly(N-isopropylacrylamide) into poly(vinyl alcohol)/ poly(sodium acrylate) (PVA/SA) hydrogel, sodium tetraborate decahydrate and poly(sodium acrylate).	A multimodal sensor is fabricated by incorporating PNIPAAm in to into PVA/SA hydrogels. Cross-linking with sodium tetraborate decahydrate and doped with poly(sodium acrylate)	lower critical solution temperature (LCST) behavior	The proposed sensor enables simultaneous touch and temperature detection with fast response time and sensitivity. Integration of LCST polymers could facilitate the development of temperature-dependent soft electronics and smart windows.	[74]
2, 2, 6, 6-tetrametylpiperidine-1-oxyl (TEMPO)-oxidized cellulose nanofibrils and a polypyrrole conductive network.	A hierarchically triple-network hydrogel is prepared using one-pot free radical polymerization.	The resulting hydrogel exhibits stretchability, self-healing, and electro-conductivity.	The hydrogel has applications in damage-free wearable electronics, real-time monitoring of human movements, and various wearable electronic applications.	[75]
2-ureido-4[11-1]-pyrimidinone (UPy), polyaniline/poly(4styrenesulfonate) (PANI/PSS) network	A multifunctional conductive polymer hydrogel is developed by incorporating multiple hydrogen-bonding and crosslinking point.	Enabling electronic conduction assisted by ionic transport.	The hydrogel finds applications in 3D printing, wearable devices, and flexible electronics, combining supramolecular chemistry with conducting polymers for advanced functional materials.	[76]
A p-n-type thermoelectric device is assembled using PEDOT:PSS and carbon nanotube fibers	Highly conductive p-type PEDOT:PSS fibers are produced through a gelation process.	Improvement of electrical conductivity and preservation of high Seebeck coefficient by post-treatment with organic solvents	The development of highly conductive PEDOT:PSS fibers enables wearable energy harvesting and advancements in wearable energy devices.	[77]
l-cysteine binder, silver nanoparticles (Ag NPs), and conductive hydrogel coating (Acrylamide (AM), acrylic acid (AA), sodium hydroxide, N,N’-methylene-bis-acrylamide (BIS), lauryl methacrylate (LMA), dodecyltrimethy lammonium bromide (DTBA), ammonium persulfate, sodium sulfite, L-cysteine and sodium borohydride)	The hydrogel prepared via a free radical copolymerization of AM, LMA, AA, and BIS in an aqueous solution in the presence of DTBA. Which is then coated on L-cysteine and Ag NPs layers (AgCy-Cot)	Remarkable electrical stability	The conductive fabric with high durability and electrical stability finds applications in smart textiles, wearable devices, and related technologies, opening up opportunities for various advancements.	[78]
Single-walled carbon nanotube and alginate	Conductive and piezoresistive spheres are embedded between conductive electrodes to create a wearable pressure sensor.	Optimization maximizes sensitivity.	The pressure sensor has applications in wrist pulse monitoring, throat muscle motion detection, and identification of external pressure distribution. It offers a low-cost and highly sensitive solution for health monitoring and human/machine interfaces.	[79]

## Data Availability

The authors partly used Open AI’s large-scale language-generation model. The authors reviewed, revised, and edited the document for accuracy and take full responsibility for the content of this publication. The authors used Bing AI image creator to draw the graphical abstract.
